# A New Mode of Treating Diseases of the Cavity of the Nose

**Published:** 1865-06

**Authors:** J. L. W. Thudichum

**Affiliations:** Lettsomian Professor of Medicine of the Medical Society of London


### A NEW MODE OF TREATING DISEASES OF THE CAVITY OF THE NOSE.

By J. L. W. THUDICHUM, M.D., M.R.C.P., Lettsomian Professor of Medicine of the Medical Society of London.

The treatment of diseases of the cavity of the nose has hitherto been attended with very great difficulties, owing to the circumstance that the cavity is large, complicated by many sinuosities, interrupted by many thin bony and membranous projections, and therefore little accessible, and for the most part not accessible at all, to instruments by which growths might be removed, or topical remedies applied. The removal of excrescences from the lower and median nasal canal was yet the most successful of surgical operations, although it was frequently left incomplete, or remained unavailing, owing to the speedy return of the polypi. But the topical application of remedies for the treatment of acute and chronic affections of the nasal cavity, which is certainly the principal therapeutic requirement, and in many cases prevents the formation of polypi, could only be attempted by mechanical contrivances which were so objectionable to the patients, that after longer or shorter trials they had to be abandoned. I have had under my own care several important cases of affection of the nasal cavity, in which the mere possibility of cleansing the cavity of the nose would have been a great boon to the patients; others, in which I have no doubt the application of remedies, such as we are in the habit of using in conjunctivitis, would have effected a speedy recovery from painful and troublesome conditions. The only mode of cleansing the cavity of the nose which was then known in medical science, was by injections with a syringe; but, owing to the velocity with which the injected fluid touched the walls of the nose, this process always created much irritation, pain, sternutation, and lachrymation, and the patients mostly opposed the entrance of the fluid by expiratory efforts, which, indeed, were the only means they had of preventing the fluids from running down the choanæ and reaching the larynx. The mere effect of pure water upon the Schneiderian membrane being highly irritating, two causes combined to defeat the object of injections of water; and when medicines which might be supposed to have a beneficial effect upon the diseased Schneiderian were dissolved in the water, they, although perhaps better tolerated than pure water, could not be kept sufficiently long in contact with the affected parts to exercise upon them even such slight medicinal action as their necessarily diluted state permitted. There was a third application that could be made—namely, the introduction of medicines in the form of fatty or mucilaginous ointments. In one case, in which I endeavored to benefit a chronic ozæna—a residue of scarlet fever—by topical applications, a solution of sulphate of zinc in intimate mixture with lard had a most decided effect, the patient being much improved, though not cured. But this application of ointment to the surface of the lower canal of the nose, and to a part of the median canal—which are the only portions that, as a rule, can be reached, even by clever manipulation—is the most objectionable of any, so far as its accompaniments of irritation and pain are concerned, sternutation and lachrymation being not rarely long continued after it, and the peculiar pain producing a reluctance on the part of the patient, which it is difficult to overcome in young and old people. All these applications, then, were partial, imperfect, irritating, and, consequently, unavailing to effect the desired end. Many cases of superficial ulceration ended in caries, embittering the life of the patients, and through the odor making intercourse impossible, and family relations troublesome; other cases of chronic inflammation ended in deformity of the external nose, and the formation of polypi in its cavity, and produced a constant false resonance of the voice; a number lasted throughout a lifetime, the nose being constantly a weak part, and capable of prostrating the patient at any opportunity which dust and wind might afford; others had consequences even more severe, and the specific ulcerations of the cavity of the nose only too frequently terminated in that sinking of its bridge which is the most painful proclamation of disease with which a patient can become afflicted. Then there were the convulsive affections produced by local irritation in the nose—those cases of fabulous sneezing in which hardly any remedy availed, even in diminishing the number of spasms in time, because the centre and seat of the irritation could not be reached by medical agencies. Truly dangerous were some cases of bleeding from the nose, in which the broken bloodvessels could not be reached by either styptics or mechanical compression, and could not be made to contract by contact with that most powerful of hæmostatic agents, ice or ice-cold water. Not a few cases of this kind terminated fatally, or required the most desperate measures to prevent the fatal end, such as plugging of the nose and choanæ with sponges or tinder; and these not rarely left a condition of anæmia in which other accidental diseases could put a stop to life with comparative ease, or which continued without the supervention of other diseases, enfeebling and considerably shortening the rest of the life of such patients.

All these difficulties, and many more which might be mentioned, are removed at one stroke, by the discovery of Professor Weber, of Halle:—that when one side of the nasal cavity is entirely filled, through one nostril, with fluid, by hydrostatic pressure, while the patient is breathing through the mouth, the soft palate completely closes the choanæ, and does not permit any fluid to pass into the pharynx (a physiological fact thus far already discovered by E. H. Weber, of Leipzig, before 1847, and published in Muller's Archiv., 1847, pp. 351–354); while the fluid easily passes into the other cavity, mostly round and over the posterior edge of the septum narium, in some persons also through the frontal sinuses, and escapes from the other open nostril, after having touched every part of the first half of the other cavity of the nose, and a great part, certainly the lower and median canal, of the second half. By means of the application of this principle to the treatment of diseases of the nose, it is possible easily and frequently to wash the nasal cavity, to disinfect and deodorize it, to remove the sordes which accumulate so easily in it, and to apply to its surface a great number of beneficial medicinal substances, so as to prevent acute affections from extending, and to incline them towards a speedy recovery; to stop hemorrhages, allay irritations, and subdue, in a remarkable manner, chronic affections of the Schneiderian membrane, so as to reëstablish a perfectly healthy surface and normal condition of the organ of smell.

*Of the medicinal solutions which may be applied to the cavity of the nose.*—Although the solutions before enumerated act in a measure as alteratives, resolvents, and escharotics, and, therefore, rarely constitute a sufficient medical application by themselves, yet they are more frequently used for preparing the nose for the application of more energetic and specifically acting solutions. To this latter class belong the solutions of alum, sulphate of zinc, and sulphate of copper—the best astringents; the solutions of nitrate of silver, and bichloride of mercury—the most suitable alteratives; and the solutions of chloride of calcium, in which suboxide or oxide of mercury is suspended in a finely subdivided state, together with the bichloride solutions —the best specifics. Of stimulating solutions, a mixture of eau de Cologne with water or salt water is sometimes useful.

The probable concentration of these solutions can be surmised from the circumstance that the sensibility of the healthy nasal cavity stands about midway between that of the eye and the mouth. When the nasal cavity is completely filled with fluid, the specific sense of smell cannot any longer be exercised; even the solution of eau de Cologne is not perceived to be such when it once fills the nose. The sense of smell being thus entirely obliterated by the fluid contained in the nose, the reflex effects which substances may exercise by means of this sense are entirely absent; and the only impingement which the fluids can produce is upon the filaments of sensitive nerves coming from the fifth pair. It is owing partly to this circumstance that comparatively strong medicinal solutions are borne by the nasal cavity without great secretion. Another circumstance favoring the application of stronger solutions, is the ready manner in which the healthy surface of the nose defends itself against irritating, chemically-impinging substances, by means of a copious flow of mucus. Excoriated or ulcerated parts lack this power of rapid secretion; and hence they are affected by medicinal solutions much more than the healthy parts of the surface of the nasal cavity. What is here stated is the general result of experience and experiment; but, at the same time, I must insist that the application of medicinal solutions in each case should be begun with the greatest caution, as individuals differ greatly in point of irritability of the nasal cavity. In the beginning, therefore, very dilute solutions of medicinal substances should be used, and their strength be increased gradually, after their effect has been well exhausted, by the use of greater quantities applied by a quick flow, or the use of smaller quantities in a slow current distributed over a longer time of contact.

*Solution of Alum.*—Half an ounce of roughly-powdered crystallized alum is dissolved in a small quantity of hot water, and the solution made up to one quart by means of cold and tepid water in such a manner as to ensure that the temperature of the solution should be below, but near to, blood-heat. In superficial ulceration, or blenorrhagic conditions, this solution is well borne. Ulcerated parts, which before its application were red, mostly appear as white patches after its application, thus showing that the effect of the alum on the ulcerated surface has been considerable. When I was desirous to manage with smaller quantities of solutions, I have sometimes mixed a little permanganate solution with that of alum.

*Solution of Sulphate of Zinc.*—From a scruple to a drachm of the sulphate of zinc, dissolved in a quart of warm water, together with half an ounce or an ounce of sulphate of soda or sulphate of magnesia, gives a suitable fluid.

*Solution of Sulphate of Copper.*—Of this sulphate also from a scruple to a drachm, mixed with half an ounce or an ounce of soda sulphate or magnesia sulphate, can be dissolved in a quart of warm water.

*Solution of Acetate of Lead.*—Of this crystallized acetate, from a drachm to two drachms, together with half an ounce or an ounce of crystallized acetate of soda, may be dissolved in a quart of warm water.

*Solution of Nitrate of Silver.*—Of this salt, not more than from half a grain to a grain should be dissolved in each ounce of water. A quart of water, therefore, in which previously from half an ounce to an ounce of nitrate of soda has been dissolved, may receive from sixteen to thirty-two grains of the nitrate. In particular cases the solution may be made stronger. The nitrate of potash is not so good as the nitrate of soda, because it has slightly irritating qualities. When it is necessary to use it in an emergency, when soda nitrate cannot be had, the solution should be more diluted.

*Solution of Bichloride of Mercury.*—The greatest caution is necessary in the use of this agent, as it has a tendency to produce excoriations on healthy surfaces. The first solution to be employed should be one containing five grains of corrosive sublimate in a quart of water, in which an ounce of common salt is also dissolved.

*Solution of Chloride of Calcium with suspended Oxide or Suboxide of Mercury.*—These fluids are the common phagedænic waters, or black and yellow wash, to which common salt has been added. Two drachms of calomel, twelve fluid ounces of lime-water, one ounce of common salt, and twenty ounces of common warm water, yield the black solution. One scruple of corrosive sublimate, one ounce of common salt, twelve fluid ounces of lime-water, and twenty fluid ounces of common warm water yield the yellow wash. These mixtures must be well agitated in the glass vessel while being allowed to run through the nasal cavity.

*Sedative solutions.*—Of prussic acid forty minims to the quart of warm salt-water, of tincture of opium two drachms may be taken. These drugs may be added to some of the above solutions of metallic salts. But if this is desired, it is better to substitute a solution of morphia for tincture of opium. The prussic acid is incompatible with the copper, silver and precipitated mercury solutions; it goes conveniently with the alum and common salt solutions.

*Styptic or hæmostatic solutions.*—Amongst these, ice-cold salt water, containing an ounce of salt to a pint of ice-water, takes the first place. When this, after having been continued for a considerable time, is insufficient to stop the hemorrhage, a fluid ounce of the tincture of sesquichloride of iron may be added to each pint of ice-cold salt-water.

*Stimulating solution.*—One ounce of eau de Cologne upon ten ounces of salt-water, is a useful stimulant. Strong spirit of wine may be taken in place of the eau de Cologne.

I have now fully, and for some readers, perhaps, somewhat too explicitly, described a number of medicinal solutions which may with advantage be applied to the treatment of nasal diseases by the method in question. I was desirous to impress upon the memory of the reader the fact that I recommend only such solutions as are brought up to a certain specific gravity by salts which do not decompose the medicinal agents. There may be cases in which it is desirable to swell the Schneiderian membrane by watery fluid and produce endosmosis, and others in which highly concentrated solutions may beneficially be used to effect exosmosis and shrivel Schneider's membrane. These adaptations, and the various accommodations of the fluids and their degrees of concentration, I must leave to the skill and ingenuity of those who make use of this method. They will, also, probably multiply the resources of the rhinotherapeutic pharmacy, and thereby add to the success and certainty of this interesting method of treatment.—*London Lancet.*

